# Effects of amodiaquine and artesunate on sulphadoxine-pyrimethamine pharmacokinetic parameters in children under five in Mali

**DOI:** 10.1186/1475-2875-10-275

**Published:** 2011-09-21

**Authors:** Mamadou M Tekete, Sékou Toure, Alfia Fredericks, Abdoul H Beavogui, Cheick PO Sangare, Alicia Evans, Peter Smith, Hamma Maiga, Zoumana I Traore, Ogobara K Doumbo, Karen I Barnes, Abdoulaye A Djimde

**Affiliations:** 1 Molecular Epidemiology and Drug Resistance Unit, Malaria Research and Training Centre, Department of Epidemiology of Parasitic Diseases, Faculty of Medicine, Pharmacy and Dentistry, University of Bamako, P.O. Box: 1805, Bamako, Mali; 2 Division of Clinical Pharmacology, Department of Medicine, University of Cape Town, Cape Town, South Africa

**Keywords:** Pharmacokinetic, Combination therapy, Sulphadoxine, Pyrimethamine, Amodiaquine, Artesunate and Malaria

## Abstract

**Background:**

Sulphadoxine-pyrimethamine, in combination with artesunate or amodiaquine, is recommended for the treatment of uncomplicated malaria and is being evaluated for intermittent preventive treatment. Yet, limited data is available on pharmacokinetic interactions between these drugs.

**Methods:**

In a randomized controlled trial, children aged 6-59 months with uncomplicated *falciparum *malaria, received either one dose of sulphadoxine-pyrimethamine alone (SP), one dose of SP plus three daily doses of amodiaquine (SP+AQ) or one dose of SP plus 3 daily doses of artesunate (SP+AS). Exactly 100 μl of capillary blood was collected onto filter paper before drug administration at day 0 and at days 1, 3, 7, 14, 21 and 28 after drug administration for analysis of sulphadoxine and pyrimethamine pharmacokinetic parameters.

**Results:**

Fourty, 38 and 31 patients in the SP, SP+AQ and SP+AS arms, respectively were included in this study. The concentrations on day 7 (that are associated with therapeutic efficacy) were similar between the SP, SP+AQ and SP+AS treatment arms for sulphadoxine (median [IQR] 35.25 [27.38-41.70], 34.95 [28.60-40.85] and 33.40 [24.63-44.05] μg/mL) and for pyrimethamine (56.75 [46.40-92.95], 58.75 [43.60-98.60] and 59.60 [42.45-86.63] ng/mL). There were statistically significant differences between the pyrimethamine volumes of distribution (4.65 [3.93-6.40], 4.00 [3.03-5.43] and 5.60 [4.40-7.20] L/kg; *p = 0.001*) and thus elimination half-life (3.26 [2.74 -3.82], 2.78 [2.24-3.65] and 4.02 [3.05-4.85] days; *p < 0.001*). This study confirmed the lower SP concentrations previously reported for young children when compared with adult malaria patients.

**Conclusion:**

Despite slight differences in pyrimethamine volumes of distribution and elimination half-life, these data show similar exposure to SP over the critical initial seven days of treatment and support the current use of SP in combination with either AQ or AS for uncomplicated *falciparum *malaria treatment in young Malian children.

## Background

Malaria remains a major public health problem, particularly in sub-Saharan Africa, where it claims nearly 750,000 lives of children under the age of five years [1]. To improve cure rates and delay the development and spread of artemisinin resistance, the World Health Organization (WHO) recommends artemisinin-based combination therapy (ACT) [2]. Nearly all malaria endemic countries and territories worldwide have adopted ACT as first-line treatment for *Plasmodium falciparum *malaria [1]. However, many studies show fewer treatment failures by day 28 when sulphadoxine-pyrimethamine (SP) is combined with amodiaquine (AQ) rather than artesunate (AS) [3-5]. SP monotherapy is the only drug recommended for intermittent preventive treatment during pregnancy (IPTp) and SP+AQ is a strong candidate for IPT in infants (IPTi) and in children (IPTc) [4-11].

Resistance to SP is wide spread in Africa, with high level of resistance in east Africa compared to west Africa. That is confirmed by the level of *dhfr *and *dhps *mutation levels, particularly *dhps540 *which best predicts the *in vivo *SP resistance [12].

Despite some level of resistance to SP and AQ, the combination of the two drugs is effective on falciparum malaria in much of West Africa.

And, despite these widespread use of SP, the pharmacokinetic parameters of SP when used in combination with AS or AQ is poorly documented, particularly in young children. Altered pharmacokinetic parameters contribute to the increased risk of young children failing SP treatment. After adjusting for dosage, median sulphadoxine and pyrimethamine areas under the concentration time curves in children aged 2-5 years old are approximately half those in adults [13]. This study investigated the effect of combination with either AQ or AS on SP pharmacokinetic parameters in children under-five years of age enrolled in a randomized controlled trial on the treatment of uncomplicated falciparum malaria.

## Methods

### Study design

This was an open label clinical trial using the WHO 2003 protocol [14] conducted in Bancoumana, Mali, during the malaria transmission seasons of 2004 (August 2004-January 2005) and 2005 (July 2005-January 2006). Bancoumana is a rural village of around 12,000 people located 60 kilometers south-west of Bamako. *Plasmodium falciparum *malaria is both endemic and seasonal with parasitaemia prevalence rates ranging from 40-50% in the dry season (October-May) and 70-85% in the rainy season (June-September)[15,16]. Inclusion criteria for this study included 1) parental consent; 2) age between 6 and 59 months; 3) an axillary temperature between 35.5°C and 39.5°C; and 4) *P. falciparum *mono-specific parasitaemia between 2,000 and 200,000 parasites/ul (parasite density was measured by microscopy after staining thick smear with Giemsa). Exclusion criteria included 1) features of severe malaria (haemoglobin < 5 g/dL; respiratory distress, renal failure; hypoglycaemia, shock; bleeding), 2) danger signs such as prostration, 3) a history of allergy or other severe adverse reaction to the study drugs and 4) more than two episodes of vomiting per day. Children with any of the above exclusion characteristics or who were considered too ill for inclusion in the study, or who declined to participate in the study, received standard and appropriate treatment with chloroquine as then recommended by the National Malaria Control Programme.

Enrolled children were randomized into three treatment arms (SP, SP+AQ or SP+AS) by computer-generated randomization. Directly observed treatment was administered based on weight to the nearest half tablet that would provide the following minimum dose(s): 25 mg/kg of sulphadoxine and 1.25 mg/kg of pyrimethamine as a single dose on day 0; with or without AQ 10 mg/kg/day over three days or artesunate 4 mg/kg/day over three days. All subjects were observed for 60 minutes to monitor for adverse reactions and to make sure that the medicine was not vomited. If vomiting occurred within 30 minutes, the full dose was re-administered. If vomiting occurred between 30 and 60 minutes a half-dose was re-administered. All acute concomitant illnesses were treated for free by the study team, with concomitant medication which had no known SP drug interactions. Parental written informed consent was obtained before any protocol specific procedure was performed. The study was conducted in accordance with the Helsinki Declaration of 1975 (as revised in 1983). The protocol was approved by the ethical committee of the Faculty of Medicine, Pharmacy and Odonto-Stomatology, Bamako, Mali.

### Study drugs quality

Study drugs (Flavoquine^® ^tablet for AQ, Fansidar^® ^tablet for SP and Artesunate tablet for AS)were bought at a private pharmacy in Bamako, Mali. The quality of these drugs were authenticated according to the United States Pharmacopoeia and National Formula (USP-NF) 2004 methods using dissolution testing apparatus in combination with High Performance Liquid Chromatography (17). More than 75% of the amount of the three drugs (sulphadoxine, pyrimethamine and amodiaquine) of the study were dissolved in the time indicated for each drug.

### Pharmacokinetic sample collection and assay

Capillary blood samples were collected by fingerprick at day 0 prior to treatment and on days 1, 3, 7, 14, 21 and 28, after SP administration into 1.5 ml Eppendorf tubes without anticoagulant. Exactly 100 μl of that blood sample was spotted onto filter paper (Whatman 3 M), air dried at room temperature and stored in plastic folders with solid desiccant until assayed in March 2008. Concentrations of sulphadoxine and pyrimethamine were determined by a validated method using liquid chromatography-mass spectrometry/mass spectrometry, with the lower limits of quantification set at 10 μg/mL for sulphadoxine and 10 ng/mL for pyrimethamine. The upper limits of quantification were set at 200 μg/mL for sulphadoxine and 1 μg/mL for pyrimethamine. The coefficient of variation for sulphadoxine was 10.3% at 65 μg/mL, and pyrimethamine had a coefficient of variation of 13.8% at165 ng/mL as previously described [13].

### Pharmacokinetic analysis

The area under the capillary blood concentration-time curve to infinity (AUC), terminal elimination half-life (t1/2), apparent volume of distribution (VD) and apparent clearance (Cl) of pyrimethamine and sulphadoxine were determined using a non compartmental model in WinNonLin Professional 3.3 (Pharsight). As there was no intravenous comparator arm in this study, equivalent bioavailability was assumed for comparisons of apparent volumes of distribution and clearance, which were expressed as Vd/f and Cl/f, where f is the fraction of drug absorbed (unknown).

### Statistical analysis

As most pharmacokinetic parameters were not normally distributed, these were summarized using medians and inter-quartile ranges and compared between the three treatment arms using the Kruskal-Wallis equality-of-populations rank test. Categorical data were compared using the Chi-squared test.

## Results

### Study population characteristics

Between 2004 and 2006, we conducted a clinical trial comparing the therapeutic efficacy of SP, SP+AQ and SP+AS. A total of 455 children were included and 441 (97%) were successfully followed for 28 days. The baseline characteristics in the three treatment arms were comparable for mean age, mean weight and gender (P > 0.05). Without PCR correction the 28-day uncorrected adequate clinical and parasitological response (ACPR) rates were above 95% for each of the treatment arms. Early treatment failure (ETF) was only found in the SP-monotherapy arm in three patients (2%, n = 147). After PCR correction by *msp2*, the ACPR rates were above 97% in all three groups.

**Figure 1:**
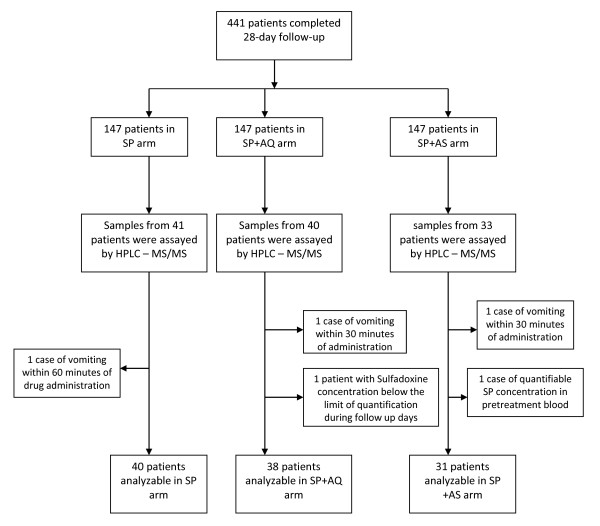
**Study profile**.

Among the study population children with complete pharmacokinetic samples from day 0 to day 28 were included in this pharmacokinetic analysis i.e. 41 children in SP arm, 40 in SP+AQ and 33 in SP+AS. The ages of the children enrolled ranged between 10 and 60 months. Treatment groups were well matched for the baseline characteristics of age, gender, weight, SP mg/kg dosage, temperature and hemoglobin (Table 1). Three patients who vomited within 60 minutes (one in each treatment arm), and one patient with quantifiable SP concentrations pre-treatment (day 0) and one patient (SP+AQ arm) in whom SP concentrations were below the limits of quantification throughout the follow up period were excluded from the analysis of pharmacokinetic parameters. This analysis was performed on a total of 40, 38 and 31 patients respectively in the SP, SP+AQ and SP+AS arms (Figure 1).

**Table 1:** Baseline characteristics [median (IQR)] by treatment group

	SP arm (n = 41)	SP+AQ arm (n = 40)	SP+AS arm (n = 33)	P-value
Age (months)	48	48	48	0.66
	(24-48)	(24-48)	(24-48)	
Gender Male	21/41	23/40	19/33	0.81
n (%)	(51%)	(58%)	(58%)	(Chi-squared)
Weight (kg)	14	13.5	13	0.93
	(11-16)	(12-16)	(11-15)	
Temperature	38.5	38.7	38.2	0.50
(Celcius)	(38.0-38.9)	(38.1-39.1)	(37.8-39.0)	
Haemoglobin	10.7	10.3	10.9	0.95
(g/dL)	(9.5-11.8)	(9.4-12.20	(8.9-11.7)	

### Pharmacokinetic analysis

**Figure 2:**
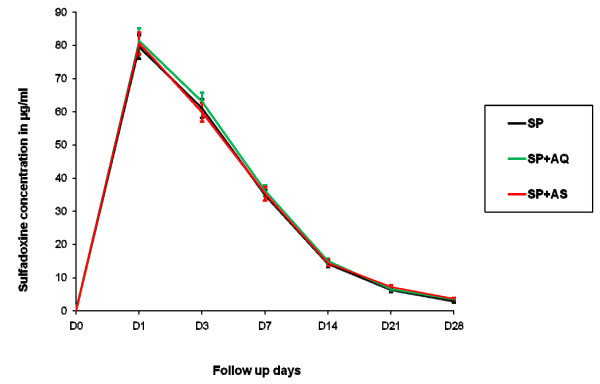
**Mean Sulphadoxine concentration profiles in young Malian children with uncomplicated falciparum malaria, by treatment arm**.

**Figure 3:**
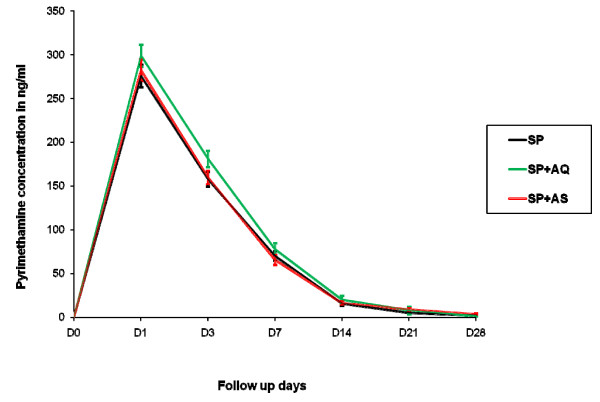
**Mean Pyrimethamine concentration profiles in young Malian children with uncomplicated falciparum malaria, by treatment arm**.

The three treatment arms had similar mean concentration time profiles of sulphadoxine (Figure 2) and pyrimethamine (Figure 3) with almost all children reaching the maximum concentration on Day 1 (103/109 (95%) for sulphadoxine and 107/109 (99%) for pyrimethamine. For sulphadoxine, the day 1, 3, and 7 concentrations as well as the AUC, apparent volume of distribution, apparent clearance and elimination half-life were similar between the three treatments arms (P > 0.05) (Table 2). For pyrimethamine, the day 1, 3, and 7 concentrations as well as the AUC and apparent clearance were similar. However, the apparent volume of distribution of pyrimethamine was lower in the SP+AQ arm when compared to SP monotherapy (p = 0.008) and SP+AS (p < 0.001) treatment arms. As a consequence the elimination half-life was lower in patients treated with SP+AQ than SP monotherapy (p = 0.046) or SP+AS (p = 0.001). The apparent Volume of distribution for pyrimethamine was also lower following SP monotherapy than SP+AS (p = 0.030) (Table 3).

**Table 2:** Sulphadoxine pharmacokinetic parameters (Median [IQR]) in Malian children under five with uncomplicated falciparum malaria, by treatment group

PARAMETERS	SP arm	SP+AQ arm	SP+AS arm	P-value [Kruskal-Wallis]
Sulphadoxine dose (mg/kg)	27.8 (25.0-27.8)	28.8 (25-31.3)	27.8 (25-28.8)	0.46
Median Concentration day 1 [IQR] (μg/mL)	73.20 [66.55-89.33]	80.95 [65.05-89.08]	76.80 [62.70-93.50]	0.86
Median Concentration day 3 [IQR] (μg/mL)	61.3 [47.8-73.0]	63.9 [49.6-72.7]	57.9 [47.4-69.8]	0.71
Median Concentration day 7 [IQR] (μg/mL)	35.25 [27.38- 41.70]	34.95 [28.60-40.85]	33.40 [24.63-44.05]	0.97
Median AUC [IQR] (μg/mL.day)	660.08 [520.37-832.11]	682.67 [579.45-797.12]	672.04 [525.35-898.86]	0.88
Median VD [IQR] (mL/kg)	349.35 [295.43-434.25]	345.60 [294.15-427.95]	357.80 [266.70-427.40]	0.97
Median T1/2 [IQR] (days)	5.60 [4.62-6.78]	5.77 [5.11-6.45]	6.13 [4.90-7.26]	0.56
Median Clearance [IQR] (mL/kg/day)	41.85 [35.13-54.78]	41.65 [34.20-52.83]	41.00 [32.10-53.40]	0.80

**Table 3:** Pyrimethamine pharmacokinetic parameters (Median [IQR]) in Malian children under five with uncomplicated falciparum malaria, by treatment group

PARAMETERS	SP arm	SP+AQ arm	SP+AS arm	P-value [Kruskal-Wallis]
Pyrimethamine dose (mg/kg)	1.39 (1.25-1.56)	1.44 (1.25-1.56)	1.39 (1.25-1.44)	0.46
Median Concentration day1 [IQR] (ng/mL)	259.50 [212.50-337.50]	300.50 [220.00-339.25]	274.00 [235.00-312.00]	0.55
Median Concentration day 3 [IQR] (ng/mL)	155 [127-187]	174 [132-215]	158 [119-191]	0.14
Median Concentration day 7 [IQR] (ng/mL)	56.75 [46.40-92.95]	58.75 [43.60-98.60]	59.60 [42.45-86.63]	0.85
Median AUC [IQR] (ng/mL.day)	1307.45 [1087.59-1773.94]	1405.32 [1110.36-2016.49]	1363.76 [1193.71-1765.91]	0.46
Median VD [IQR] (L/kg)	4.65 [3.93-6.40]	4.00 [3.03-5.43]	5.60 [4.40-7.20]	0.001
Median T1/2 [IQR] (days)	3.26 [2.74-3.82]	2.78 [2.24-3.65]	4.02 [3.05-4.85]	0.0016
Median Clearance [IQR] (L/kg/day)	1.10 [0.80-1.38]	1.00 [0.70-1.30]	1.00 [0.80-1.20]	0.38

For VD: SP VS. SP + AQ, *P = 0.008*; SP + AQ VS. SP + AS, *P = 0.000; *and SP VS. SP +AS, *P = 0.030*

For T1/2: SP VS. SP +AQ, *P = 0.046*; SP + AQ VS. SP + AS, *P = 0.001; *and SP VS. SP + AS, *P = 0.071*

As previously reported this study showed relatively wide inter-individual variation with Coefficients of variation of 29.8% and 48.7% for Day-7 concentrations of sulphadoxine and pyrimethamine, respectively.

The child who was retreated with a half-dose following vomiting between 30 and 60 minutes had SP concentrations similar to the medians on day 1 (77.7 μg/ml for sulphadoxine and 234 ng/ml for pyrimethamine). The two children retreated with the full dose for vomiting within 30 minutes had SP concentrations on day 1 that were almost double the median (133 and 137 μg/ml for sulphadoxine and 513 and 661 ng/ml for pyrimethamine). There were no serious adverse events during this study.

## Discussion

This study reports on SP pharmacokinetic parameters in young African children with uncomplicated *P. falciparum *malaria in the context of combination therapies, and confirmed the lower SP concentrations previously reported for this age group when compared with adult malaria patients [13]. There was no statistically significant difference between sulphadoxine and pyrimethamine concentrations (at day 1, day 3 and day 7), AUC and elimination half-life between the SP monotherapy and SP+AS arms. AS having no impact on these SP pharmacokinetic parameters is consistent with a study previously reported in healthy adults when AS was administered concomitantly with SP [18] and is reassuring, supporting the use of AS in combination with SP. However, a higher volume of distribution of pyrimethamine in the SP + AS arm when compared to SP monotherapy arm was observed in this study [median 5.60 vs. 4.65 L/kg; p = 0.030 (Table 3)].

Although adding amodiaquine to SP had no effect on sulphadoxine pharmacokinetic parameters, a significant decrease in the apparent volume of distribution and elimination half-life of pyrimethamine in the SP+AQ arm was observed. However, this did not result in a significant change in pyrimethamine concentrations in the blood in SP+AQ arm over the critical initial seven days of treatment nor the AUC. The absence of a substantial PK interaction in our study population supports the effectiveness of SP+AQ in IPTc [7,10] and in uncomplicated falciparum malaria treatment in children under five [5]. The effect of the shorter pyrimethamine elimination half-life could possibly reduce the post-treatment prophylactic effect of sulphadoxine-pyrimethamine plus amodiaquine as resistance to these partner drugs increases.

Day-7 concentrations of sulphadoxine and pyrimethamine are a good surrogate measure of their respective total whole blood AUCs [13] and Day 7 concentration is a predictor of treatment outcome [19]. Day7 pyrimethamine concentration observed in this study was slightly higher in SP+AQ arm, but this difference was not statistically significant (Figure 3). When compared with SP Day-7 capillary blood concentrations previously published for children aged 2-5 years in southern Africa [sulphadoxine median 31.3 (IQR 19.7-52.0); pyrimethamine median 70.3 (IQR 39.0-101.9)],[13] day-7 concentrations in young Malian children were similar for sulphadoxine, but appeared lower for pyrimethamine.

The vast majority of our patients achieved maximum concentrations of both sulphadoxine and pyrimethamine on day 1. However, the exact Cmax and Tmax could not be determined precisely because of limited sampling frequency. The concentration of artesunate could not be quantified because of the sampling methodology used, and the concentrations and pharmacokinetic parameters of amodiaquine (when administered with SP) will be reported separately.

The two children who were retreated with the full dose after vomiting within 30 minutes of SP administration had approximately double the median SP concentrations on day 1, suggesting that further data is needed to inform optimal dosing in patients with early vomiting.

In this study conducted in Mali in 2004-2006, SP or its combination with AS or AQ remained highly effective with more than a 95% adequate clinical and parasitological response rate. Although this level of efficacy of SP when administered alone or in combination with either AS or AQ is quite high in comparison with data from Eastern or Southern Africa [20,21], these rates are common in West Africa [22-24] and may reflect the lower frequency of dihydrofolate reductase (dhfr) and dihydropteroate synthetase (dhps) mutations associated with SP resistance [25,26]. Probably because of their strong synergy, the minimum effective concentrations (MEC) of sulphadoxine and/or pyrimethamine have not been clearly defined. In Gabon, where the prevalence of DHFR triple mutation was ~70%, day 3 concentrations associated with treatment success were 100 μg/mL for sulphadoxine and 175 ng/mL pyrimethamine [27]. Of the 109 children in this study, only 4 (3.7%) and 40 (36.7%) achieved these 'therapeutic' day-three concentrations of sulphadoxine and pyrimethamine, respectively. This might suggest that as *dhfr/dhps *mutations accumulate the SP concentrations achieved may be insufficient to ensure an adequate clinical and parasitological response rate as recently described in Malawi [28].

## Conclusion

This pharmacokinetic data supports the current use of SP in combination with AS to treat young Malian children with uncomplicated falciparum malaria, and the use of SP + AQ for IPTc. Ongoing monitoring is essential, particularly given the lower pyrimethamine concentrations and the accumulation of *dhfr *and *dhps *mutations that typically occurs in areas with large-scale deployment of SP.

## Conflict of interest

The authors declare that they have no competing interests.

## Authors' contributions

MMT contributed to the study design, field studies, pharmacokinetic analyses, data analysis and drafted the manuscript. AHB, CPOS, HM and ZIT, conducted the field studies, collected the samples, and assisted with manuscript preparation. PS, AF and AE developed and or conducted pharmacokinetic assay. KIB contributed to data analysis and manuscript writing. OKD contributed to study design and manuscript writing. AD contributed to the design of the study, oversaw the field and laboratory studies, and to the writing and final approval of the manuscript. All authors read and approved the final manuscript.
